# Genetic Diversity, Population Structure and Evidence of Genetic Bottleneck in *Geoffroea decorticans* (Fabaceae): Implications for Conservation

**DOI:** 10.1002/ece3.73527

**Published:** 2026-04-29

**Authors:** Roberto Contreras, Mariana Arias‐Aburto, Cecilia Bessega

**Affiliations:** ^1^ Centro Regional de Investigación y Desarrollo Sustentable de Atacama (CRIDESAT) Universidad de Atacama Copiapó Chile; ^2^ Núcleo Milenio de Ecología Histórica Aplicada Para los Bosques Áridos (AFOREST) Santiago Chile; ^3^ Departamento Ecología, Genética y Evolución (EGE), Facultad de Ciencias Exactas y Naturales Universidad de Buenos Aires Buenos Aires Argentina; ^4^ Instituto de Ecología, Genética y Evolución (IEGEBA) CONICET‐Universidad de Buenos Aires Buenos Aires Argentina

**Keywords:** arid landscape, Atacama, *Chañar*, heterozygote excess, microsatellites, SSR

## Abstract

*Geoffroea decorticans* is a drought‐tolerant native tree to the Atacama Desert, one of the most extreme hyper‐arid environments on Earth. Despite its ecological and socioeconomic value, little is known about its genetic status across a fragmented distribution. This study aimed to characterize the genetic diversity and population structure, and to assess evidence of genetic bottlenecks to propose conservation strategies for long‐term persistence. We genotyped 194 individuals from eight natural populations spanning the species' range in northern Chile using nine nuclear microsatellite *loci*. We analyzed genetic diversity, differentiation, and structure. In addition, bottleneck signatures were evaluated through heterozygosity excess, allele frequency distribution, and M‐ratio tests, and the effective population size (Ne) was estimated for each population. Null‐allele frequencies were low across the studied *loci*. Most populations exhibited moderate to high levels of genetic diversity (*Ar* = 3.86, *H*_O_ = 0.73, *H*_E_ = 0.625) and significant heterozygote excess (*F*_IS_ = −0.192). Pairwise linkage disequilibrium patterns across *loci* varied among populations, and we detected significant genetic structure and differentiation. Multiple populations showed evidence of recent and/or historical bottleneck events, with low effective population sizes (N_E‐LD_ < 20). We documented genetic variation, population structure, and bottleneck signals in *G. decorticans* populations across the Atacama Desert and emphasize the urgency of genetic monitoring. Conservation plans should prioritize populations based on genetic information including those with low diversity, strong bottleneck signals and high uniqueness; promote rigorous evaluation of source materials for future assisted gene flow programs, and protect habitat corridors to maintain adaptive potential under increasing aridification.

## Graphical abstract

We documented genetic variation, population structure, and bottleneck signals in *Geoffroea decorticans* (Fabaceae) populations across the Atacama Desert and emphasize the urgency of genetic monitoring. Conservation plans should prioritize populations based on genetic information including those with low diversity, strong bottleneck signals and high uniqueness; promote rigorous evaluation of source materials for future assisted gene flow programs, and protect habitat corridors to maintain adaptive potential under increasing aridification.

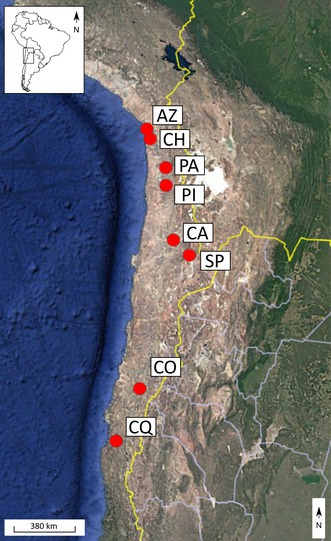

## 1 Introduction

The genus *Geoffroea* Jacq. (Fabaceae) includes several South American species, among which *G. decorticans* (Gillies ex Hook. & Arn.) Burkart, commonly known as *chañar*, is one of the few native trees adapted to the hyper‐arid Atacama Desert. From an economic perspective, it is used for food, construction and medicinal purposes. The wood is employed in the manufacture of artifacts, furniture, and housing; the fruits can be consumed fresh or dried and are also used to make flour and alcoholic beverages (Giménez 2004; Núñez et al. 2009; Castro 2020; Ugalde et al. 2020). The bark, leaves and flowers possess medicinal properties (Costamagna et al. 2013; Echeverría et al. 2020); the seeds show anticoagulant and hypoglycaemic activity (Cotabarren et al. 2020) and have notable nutritional properties (Maestri et al. 2001; Cittadini et al. 2021).

Although *G. decorticans* is globally classified as Least Concern by the IUCN, in Chile it has been classified as Vulnerable, reflecting evidence of a historical reduction in its area of occupancy and ongoing pressures from land‐use change, agriculture, and extraction for wood and firewood (Groom 2012; MMA 2021). As one of the few native woody legumes of the Atacama Desert, *G. decorticans* plays an important ecological role by contributing to habitat provision, food availability, and soil enrichment through biological nitrogen fixation, thereby supporting ecosystem functioning in extremely dry environments (Contreras et al. 2023). Conserving this species is therefore important not only to prevent further local population decline, but also to preserve the ecological functions it supports in hyper‐arid desert systems. In this context, maintaining genetic diversity is especially important because it underpins adaptive potential and long‐term persistence under increasing aridification and continued pressure on scarce water resources associated with human activities, including agriculture, urban demand, and mining (MMA 2021; Contreras et al. 2023).

In this scenario, the spatial arrangement of populations becomes a critical factor. Considering its geographical distribution, natural populations of *G. decorticans* in and around the Atacama Desert constitute an informative study system, as connectivity among populations depends on dispersal capacity and landscape barriers. C*hañar* occurs in small, discontinuous groves across the Atacama Desert, specifically in valleys, ravines, oases and near human settlements; generally, where groundwater and fog are present (Contreras et al. 2018, 2019). The effects on landscape isolation may resemble those observed in forested populations under anthropic pressures (urbanization, overexploitation, and conversion to cropland), including habitat fragmentation, shifts in mating system, and restricted gene flow (Cascante et al. 2002; Jump and Penuelas 2006; Bessega et al. 2021). Populations that become genetically isolated risk losing the genetic diversity critical for long‐term persistence (Sork and Smouse 2006). As a consequence of isolation, loss of alleles and reduction in population size are expected, leading to inbreeding, increased population divergence, and reduced within‐patch genetic diversity (Lowe et al. 2005).

Reductions in effective population size (Ne) resulting from climatic shift, habitat fragmentation, and population isolation have been widely documented in forest trees (Ledig et al. 1999; Al‐Rabab'ah and Williams 2004; Stefenon et al. 2008, 2016). Such effective population size declines can have severe negative consequences, including losses of adaptive and reproductive potentials and elevated extinction risk (Nei et al. 1975) through the combined effect of demographic, genetic, and environmental stochasticity (Luijten et al. 2000). Populations with declined effective size are especially vulnerable to genetic drift, which promotes allele fixation and the erosion of genetic variation (Luijten et al. 2000), thereby increasing differentiation among populations and generating inbred progeny. This progeny, enriched for homozygous genotypes identical by descent, is prone to inbreeding depression (Keller and Waller 2002). Inbreeding depression negatively affects fitness across the plant life cycle (Finkeldey and Hattemer 2007; Cheptou 2024), diminishing reproductive output and, consequently, adaptive capacity. Therefore, management and conservation programs for threatened, ecologically important, or commercially valuable plant species should incorporate genetic monitoring because reduction in effective size may occur without being detected by standard demographic monitoring alone (Stefenon et al. 2016).

To address these conservation concerns and understand the genetic consequences of isolation in *G. decorticans* , molecular tools can be used. Microsatellite markers are particularly well‐suited for this study due to their high polymorphism, codominant inheritance, and reproducibility, making them ideal for assessing fine‐scale genetic diversity and bottleneck signals in non‐model species (Selkoe and Toonen 2006). A previous study by Contreras et al. (2019) cross‐amplified five microsatellite *loci* from *G. spinosa* in *G. decorticans* populations of the Atacama Desert to assess genetic variability and differentiation. In broad terms, they reported high genetic diversity, pronounced differentiation and moderate gene flow among populations. That work can be considered preliminary, as no species‐specific microsatellite primers for *G. decorticans* had been developed by 2021 (Contreras et al. 2021). In addition, signals of genetic bottlenecks had not previously been evaluated in *G. decorticans. Here* we sampled 194 individuals from eight natural populations spanning northern Chile (Arica y Parinacota to Coquimbo) to conduct a comprehensive analysis of genetic diversity, population structure and bottleneck signals using microsatellite markers. Our objectives were to (1) characterize the overall pattern of genetic diversity and structure within and among populations; (2) examine whether populations exhibit molecular evidence of genetic bottleneck events; and (3) provide genetic data to inform evidence‐based conservation strategies for sustainable management of this species. We discuss our results in the context of earlier work and derive recommendations for resource conservation.

## 2 Material and Methods

### 2.1 Plant Material, Sampling and DNA Extraction

**FIGURE 1:**
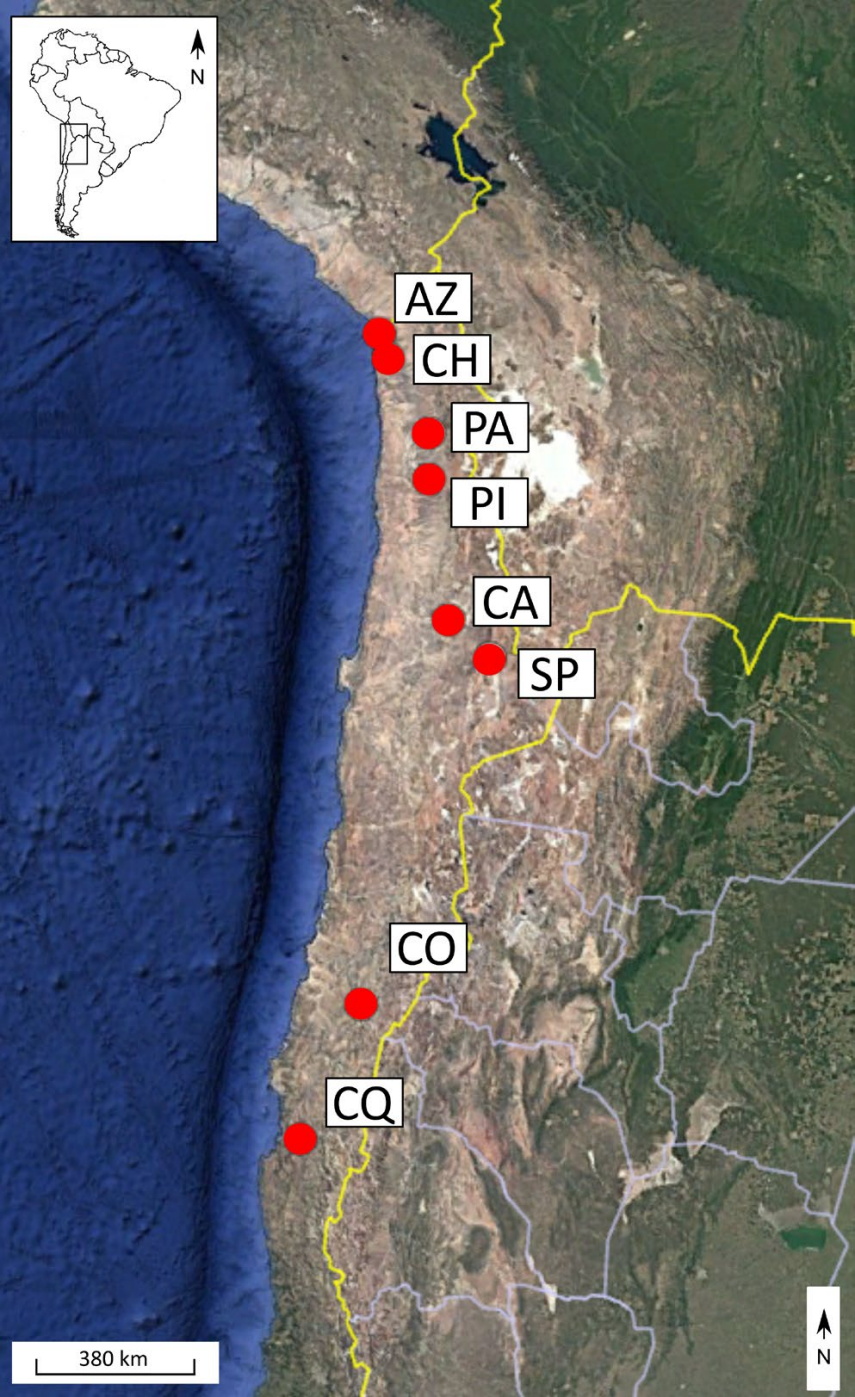
Geographic location of the eight collection sites of *G. decorticans* studied from Chile. AZ, Azapa; CA, Calama; CH, Chaca; CO, Copiapo; CQ, Coquimbo; PA, Pachica; PI, Pica; SP, San Pedro. AZ and CH from Arica y Parinacota, PA and PI from Tarapacá, CA and SP from Antofagasta, CO from Atacama and CQ from Coquimbo Region, respectively. Color map taken from Google Earth.

The sampling range extended from 18° to 27° S from Arica‐Parinacota to Coquimbo, Chile (Figure 1). We sampled eight natural populations of *G. decorticans* to capture the most representative populations of the Atacama Desert. Fresh young leaves were collected from trees spaced ≥ 30 m apart to minimize sampling of close relatives. Based on our sampling criteria and population sizes, 7–42 adult trees were sampled per site, for a total of 194 adults. Herbarium vouchers were deposited in the Herbarium of the Departamento de Silvicultura y Conservación de la Naturaleza, Universidad de Chile (EIF), under the voucher's numbers: EIF13800, EIF13803, EIF13807, EIF13809, EIF13812 and EIF13815. Fieldwork was challenging due to the geographic and environmental conditions at several sites, including difficult access to certain valleys, high temperatures, intense solar radiation, and large inter‐individual distances. Geographical coordinates were recorded for each sampled tree using GARMIN ETREX 10 GPS RECEIVER.

Genomic DNA was isolated from fresh leaf tissue of 194 individuals using a modified cetyltrimethylammonium bromide (CTAB) protocol (Contreras et al. 2019).

### 2.2 Microsatellite *Loci* Selection

Genomic resources for *G. decorticans* were previously established by Contreras et al. (2021), who identified 144,117 microsatellite *loci* via Illumina next‐generation sequencing (NGS). Leaf material from 30 individuals across five Chilean regions (Arica‐Parinacota, Tarapacá, Antofagasta, Atacama, and Coquimbo) was pooled for CTAB‐based DNA extraction and library preparation. Following sequencing (Genoma Mayor, Chile), SSRs were screened using MISA (Thiel et al. 2003). Primers were subsequently designed with Primer3 (Rozen and Skaletsky 2000) using the following criteria: 90–230 bp product size, 52°C–62°C melting temperature (Tm), and 30%–79% GC content. This pipeline led to the formal description and evaluation of the first 41 SSR primer pairs.

From these 41 SSR *loci* previously characterized for *G. decorticans* , six markers (SSRGD27558, SSRGD11258, SSRGD7345, SSRGD17951, SSRGD11733, and SSRGD17837) were initially selected at random for inclusion in this study. To ensure a representative sample of the genome and avoid systematic bias, this set was subsequently augmented with three additional markers (SSRGD8699, SSRGD8997, and SSRGD13517) chosen from the available SSR sequences derived from the same Illumina run (deposited in the SRA under BioProject ID PRJNA719569 and BioSample SAMN18613292, Table S1). This selection strategy was specifically designed to achieve a balanced distribution of the most abundant repeat types (di‐ and tri‐nucleotides), resulting in a final panel of nine *loci* that provide a good representation of the species' genetic variation while minimizing stuttering effects (Selkoe and Toonen 2006; Guichoux et al. 2011). All forward primers were fluorescently labeled with TAM and HEX synthesized by Macrogen Inc. (https://dna.macrogen.com/) (Table S1).

### 2.3 Microsatellite Genotyping

PCR amplifications were performed in single‐plex reactions to ensure optimal amplification and avoid potential primer‐primer interactions. Each 24 μL reaction contained 20 ng genomic DNA, 0.3 μM of each primer, and 12 μL SapphireAmp Fast PCR Master Mix 2X (TAKARA, Clontech). Negative controls, containing all reagents except template DNA, were included in every PCR run to monitor for potential contamination. Reactions were run on a MultiGene OptiMax Thermal Cycler (Labnet, USA) with the following program: 94°C for 3 min, followed by 40 cycles of 94° for 25 s, primer‐specific annealing at 55°–60° (Table S1) for 25 s, and 72°C for 25 s; followed by a final extension at 72°C for 6 min. PCR products were analyzed on an ABI3730XL Genetic Analyzer (Applied Biosystems), and allele sizes were determined with Peak Scanner v1.0 (Applied Biosystems, CA, USA). Regarding the scoring criteria, a successful amplification was defined by the presence of clear, reproducible peaks with signal intensities above 200 relative fluorescence units (RFU). To ensure data completeness and rule out potential pipetting errors, PCR amplifications that initially failed were repeated.

### 2.4 Genetic Diversity

Linkage disequilibrium was evaluated using the method implemented in the *test_LD* function of the *Genepop* package (Rousset 2008) in R 4.4.0 (R Core Team 2025). The significance of genotype independence was estimated via a Markov Chain Monte Carlo (MCMC) algorithm. Following the pairwise tests, a False Discovery Rate (FDR) correction (Benjamini and Hochberg 1995) was applied to account for multiple comparisons.

Null allele frequencies were estimated for each locus using the Maximum Likelihood approach implemented in ML‐NullFreq software (Kalinowski and Taper 2006). The statistical significance was assessed using 10,000 Monte Carlo randomizations as described by Guo and Thompson (1992) and the U‐test statistics described by Rousset and Raymond (1995). Small *p* values are interpreted as indicating a deficiency of heterozygotes, which may be due to the presence of one or more null alleles at the locus. Genetic diversity for each population was quantified as the number of observed alleles (A), the mean percentage of total alleles observed per population (*%TA*), allelic richness (*Ar*), observed heterozygosity (*H*_O_), and expected heterozygosity corrected for sample size (*H*_E_) (Nei 1978) using the *divBasics* function in the *diveRsity* package (Keenan et al. 2013) for R software. Homozygote excess/deficiency was evaluated by the fixation index (*F*_IS_) and the statistical significance of *F*_IS_ was evaluated from 95% confidence intervals obtained by 2000 bootstrap resamples.

### 2.5 Population Differentiation and Structure

Population differentiation from microsatellites data was quantified using *F*_ST_ coefficient with the *varcomp.glob* function in *hierfstat* (Goudet 2005) in R. Significance was assessed by permutation with the *test*.g function (2000 permutations).

To test isolation by distance (IBD), a pairwise genetic distance matrix was compared with the corresponding geographic‐distance matrix using a Mantel test with 2000 replicates. No transformations were applied to either matrix, and the test was one‐tailed as a positive correlation was expected. The analysis was performed using the function *mantel.rtest* in *ade4* (Chessel et al. 2004; Dray and Dufour 2007, Bougeard and Dray 2018). Pairwise genetic distances were computed as *F*_ST_ between populations with the *pp.fst* function of the package *hierfstat* in R. Geographic distances were calculated from sampling coordinates. Pairwise *F*_ST_ estimates were visualized as a heat map using the *levelplot* function of the *lattice* package (Sarkar 2008).

Molecular variance distribution was evaluated by AMOVA using Φ statistics with *poppr* (Kamvar et al. 2014, 2015) to estimate differentiation among populations, among individuals within populations, and within individuals. Significance of Φ statistics was obtained by permutation with *randtest.amova* function of the *ade4* package (Chessel et al. 2004; Dray and Dufour 2007; Bougeard and Dray 2018), with 2000 permutations.

Genetic differentiation among populations was evaluated with a Neighbor‐Joining (NJ) tree based on Nei's genetic distance, generated with *aboot* function in *poppr* package and supported by 1000 bootstrap replicates. Genetic structure was further evaluated by discriminant analysis of principal components (DAPC; Jombart et al. 2010), using *dapc* function in *adegenet* package (Jombart 2008; Jombart and Ahmed 2011) with *prior* grouping by population.

### 2.6 Bottlenecks and Population Size

We assessed signatures of reduced population size using three different approaches. The first method consists in testing for each population sample and for each locus heterozygosity excess in comparison to predictable heterozygosity under mutation drift equilibrium considering the number of alleles (Cornuet and Luikart 1997). To determine whether populations exhibit a significant excess across *loci*, we applied the sign test, the standardized difference test (Cornuet and Luikart 1997), and the Wilcoxon signed‐rank test (Luikart and Cornuet 1998) under the Infinitesimal Allele Model (IAM), the Two‐Phases Mutation Model (TPM), and the Stepwise Mutation Model (SMM). The second method assumes that populations experienced a recent reduction in effective size tend to show a shift in the allelic frequency distribution, wherein the proportion of low‐frequency alleles (< 0.1) is reduced relative to intermediate allele frequency classes (Luikart et al. 1998). Bottlenecked populations are expected to display a mode‐shifted distribution, whereas populations that did not suffer reduction in effective size typically show an L‐shaped distribution. The third method comprises the analysis of the ratio of the total number of alleles (*k*) to the overall range in size (*r*) as *M* = *k*/*r* (Garza and Williamson 2001). *M* values lower than 0.68 have often been used as a general indicator of ancient and severe bottleneck signals; thus, this threshold was adopted here, although it depends on mutation model parameters (Garza and Williamson 2001; Williamson‐Natesan 2005). The first and second methods were conducted in BOTTLENECK v1.2.02 (Piry et al. 1999), and for the third, we used the *mRatio* function in the *stratag* package (Archer 2016) in R.

We estimated effective population size using the linkage disequilibrium method (LDNe) of Hill (1981) as implemented in NeEstimator v2.1 (Waples and Do 2008; Do et al. 2014), which includes the bias correction of Waples (2006). For Ne_LD_, we set Pcrit = 0.02, which provides a good balance between accuracy and bias (Waples and Do 2008). Confidence Intervals for Ne_LD_ were obtained from the scaled *X* ^2^ distribution implemented in the software.

## 3 Results

### 3.1 Genetic Diversity

Across all populations, highly significant linkage disequilibrium (LD) was detected in all the locus pair according to the exact tests for genotypic LD between pairs of *loci* implemented in *Genepop* package (*p* < 0.001). The proportion of significant pairwise comparison was 66.9% (Table S2) after FDR correction. To test whether the observed LD could be attributed to physical linkage, we examined the consistency of pairwise LD patterns across populations (Table S2). We found no consistent pattern suggesting that the genome‐wide LD is more likely driven by demographic processes (e.g., population structure, bottlenecks) than by physical linkage among *loci*.

The presence of null alleles resulted significant (*p* < 0.05) in 9 out of 72 locus‐population combinations (12.5%), being the null allele frequencies greater than 0.10 in only 3 cases (4.1%; Table 1). As the occurrence of null alleles was low in general terms and random with respect to population and geography, the impact of null alleles was considered minimal and therefore all nine *loci* were retained for downstream analyses.

**TABLE 1:** Frequency of null alleles estimated, and significance (*p* val. in brackets) obtained with the software ML‐Null (Kalinowski and Taper 2006).

	SSRGD27558	SSRGD11258	SSRGD7345	SSRGD17951	SSRGD11733	SSRGD8699	SSRGD8997	SSRGD17837	SSRGD13517
Azapa	0.036 (0.064)	0.000 (0.484)	0.000 (1.000)	0.000 (0.492)	0.000 (0.986)	0.000 (1.000)	0.000 (0.515)	0.000 (1.000)	0.000 (0.999)
Calama	0.000 (0.077)	0.194 (0.031*)	0.000 (0.859)	0.000 (0.330)	0.000 (0.579)	0.000 (0.079)	0.082 (0.026*)	0.000 (0.961)	0.000 (0.911)
Chaca	0.000 (0.734)	0.000 (0.877)	0.000 (0.641)	0.011 (0.297)	0.000 (0.433)	0.000 (0.998)	0.021 (0.256)	0.000 (0.917)	0.000 (0.996)
Copiapo	0.040 (0.105)	0.028 (0.138)	0.000 (0.896)	0.018 (0.050)	0.044 (0.003*)	0.000 (0.838)	0.038 (0.061)	0.000 (0.286)	0.000 (0.531)
Coquimbo	0.000 (0.994)	0.000 (0.315)	0.000 (1.000)	0.000 (1.000)	0.077 (0.081)	0.000 (0.969)	0.107 (0.042*)	0.019 (0.272)	0.000 (0.708)
Pachica	< 0.001 (0.000**)	0.000 (0.630)	0.000 (1.000)	0.000 (1.000)	0.000 (1.000)	0.000 (1.000)	0.000 (0.876)	0.000 (1.000)	0.000 (0.891)
Pica	0.000 (0.158)	0.101 (0.012*)	< 0.001 (0.000**)	0.000 (0.05)	0.000 (0.968)	0.000 (0.052)	0.000 (0.995)	0.000 (0.67)	0.000 (0.991)
San Pedro	0.000 (0.042)	0.000 (0.435)	0.000 (0.734)	0.000 (0.600)	0.042 (0.003*)	0.000 (0.782)	0.027 (0.095)	0.051 (0.009*)	0.000 (0.776)

* *p* < 0.05.

** *p* < 0.01.

The estimates of the genetic diversity for *G. decorticans* in the eight populations are shown in Table 2. A mean total of 48 alleles was obtained from nine microsatellite *loci* across all populations. Allelic richness (*Ar*) ranged from 2.55 to 5.63, and the mean percentage of total alleles observed per population (*%TA*) ranged from 24.88 to 63.27. The population with the highest *Ar* and *%TA* was copiapo, whereas pachica had the lowest values. The observed (*H*_*O*_) and expected heterozygosity (*H*_*E*_) ranges from 0.6 to 0.81 and 0.47 to 0.77, respectively. Overall, *G. decorticans* showed a moderate‐high level of genetic diversity (mean *H*_*O*_ = 0.73). Significant deviations from Hardy–Weinberg equilibrium (HWE) were detected in 23 of 72 population‐locus comparisons (32%; Table S3) after applying Bonferroni correction for multiple tests. The pattern of deviation was population‐specific: most *loci* deviated from HWE in PACHICA and COQUIMBO, whereas most *loci* were in HWE in CHACA. The inbreeding coefficient estimates (*F*_IS_) were negative in all populations ranging from −0.600 to −0.006. *F*_IS_ was considered significantly different from zero in six populations (azapa, calama, chaca, coquimbo, PACHICA and PICA) as the confidence interval CI_95%_ did not overlap zero (Table 2).

**TABLE 2:** Genetic diversity indices in the eight *Geoffroea decorticans* populations based on 9 SSR *loci*.

	*N*	*A*	*%TA*	*Ar*	*Ho*	*H*_*E*_	*F*_*IS*_	[CI_*95%*_]
Azapa	42.00 (0.00)	51.00 (0.577)	41.57 (3.637)	4.330 (0.409)	0.810 (0.058)	0.700 (0.037)	−0.149*	[−0.201–0.100]
Calama	7.00 (0.00)	32.00 (0.444)	27.94 (5.478)	3.240 (0.362)	0.680 (0.110)	0.580 (0.067)	−0.176*	[−0.506–0.060]
Chaca	23.00 (0.00)	48.00 (0.471)	41.19 (5.617)	4.090 (0.313)	0.760 (0.051)	0.690 (0.028)	−0.114*	[−0.183 to −0.054]
Copiapo	32.00 (0.00)	82.00 (1.419)	63.27 (5.338)	5.630 (0.546)	0.780 (0.031)	0.770 (0.039)	–0.007^**NS**^	[−0.062–0.049]
Coquimbo	19.00 (0.00)	47.00 (0.572)	37.90 (3.096)	4.160 (0.290)	0.780 (0.071)	0.690 (0.041)	−0.138*	[−0.216–0.081]
Pachica	25.00 (0.00)	31.00 (0.580)	24.88 (3.625)	2.550 (0.300)	0.800 (0.112)	0.500 (0.068)	−0.600*	[−0.721–0.488]
Pica	10.00 (0.00)	34.00 (0.547)	26.75 (3.199)	2.890 (0.363)	0.630 (0.132)	0.470 (0.086)	−0.344*	[−0.747 to −0.099]
San Pedro	36.00 (0.00)	59.00 (1.192)	45.70 (4.673)	3.990 (0.524)	0.600 (0.078)	0.600 (0.081)	–0.006^**NS**^	[−0.067–0.049]
Mean	24.25 (0.00)	48.00 (0.725)	38.65 (4.333)	3.860 (0.388)	0.730 (0.080)	0.625 (0.056)	−0.192 (0.069)	—

Abbreviations: *%TA*, mean percentage of total alleles observed per population; *A*, observed alleles; *Ar*, allelic richness; *F*_*IS*_, inbreeding coefficient; *F*_*IS*_ was considered significant (*) when zero is not included in 95% confidence intervals [CI_95%_]; *H*_*E*_, expected heterozygosity corrected for sample size (Nei 1978); *H*_*O*_, observed heterozygosity; *N*, mean number of individuals analyzed per locus; SE in parenthesis.

### 3.2 Population Differentiation and Structure

*F*_ST_ analysis indicated relatively high and highly significant genetic differentiation among the eight populations (*F*_ST_ = 0.208, *p* = 0.001). Although the analysis was conducted using non‐transformed matrices (which requires cautious interpretation), the mantel test revealed that *F*_ST_ and geographic distance matrices were not significantly correlated (*r* = −0.23; *p* = 0.795), suggesting no association between genetic differentiation and geographical distribution.

**FIGURE 2:**
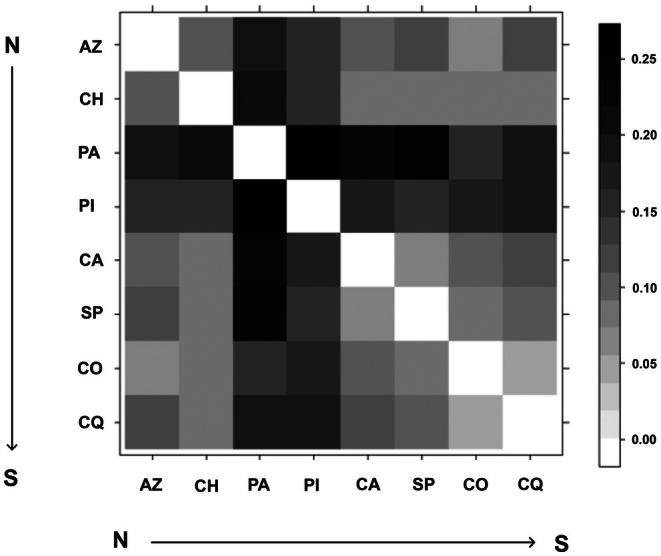
Heat map of *F*_*ST*_ pairwise comparisons between populations. White cells are low F_ST_ values; darker cells are increasingly higher F_ST_. AZ, Azapa; CA, Calama; CH, Chaca; CO, Copiapo; CQ, Coquimbo; PA, Pachica; PI, Pica; SP, San Pedro.

Pairwise differentiation indices showed considerable variation among population pairs. Overall, *F*_ST_ ranged from 0.06 to 0.39; in some cases, *F*_ST_ estimates were lower than 0.1 (CALAMA‐SAN PEDRO and COPIAPO‐COQUIMBO), while in others (PACHICA‐PICA, PACHICA‐CALAMA and PACHICA‐SAN PEDRO, PACHICA‐CHACA, PACHICA‐COQUIMBO) the estimates were approximately three times higher (> 0.3). The heatmap (Figure 2) based on pairwise *F*_ST_ likewise indicates no clear isolation‐by‐distance pattern. For example, differentiation between PACHICA and PICA (both populations in Tarapacá region) was among the highest observed (*F*_ST_ = 0.39; black in the heatmap) and similar to that between PACHICA and SAN PEDRO (*F*_ST_ = 0.40), despite the latter pair being much farther apart (Antofagasta vs. Tarapacá regions). In addition, the two most distant populations (AZAPA and COQUIMBO) did not show the greatest differentiation (*F*_ST_ = 0.19). In the same way, PACHICA appeared highly differentiated from most other populations, yet was not strongly differentiated from COQUIMBO (from Coquimbo region).

AMOVA results (Table 3) showed that most genetic variation resided within individuals (89.77%). For the remaining, a slightly negative variance component was obtained for the among‐individuals‐within‐populations level. Negative variance components are statistical artifacts and are conventionally interpreted as zero, indicating that no significant genetic structure was detected at this level beyond that already explained by the among‐population component, which was highly significant (Φ = 0.102, *p* = 0.0005).

**TABLE 3:** Analysis of molecular variance (AMOVA) based on 9 SSR *loci* considering populations and individuals.

Source of variation	df	SSD	MSD	Est var	%	*Φ*	*p*
Between populations	7	527.72	75.39	1.49	20.07	0.102	0.0005
Between samples within populations	186	969.94	5.21	−0.73	−9.84	−0.123	1
Within samples	194	1296.00	6.68	6.68	89.77	0.201	0.0005
Total	387	2793.66	7.22	7.44	100		

Abbreviations: %, proportion of genetic variance; df, degrees of freedom; Est Var., estimated variance; MSD, mean squares; *p*, significance level; SSD, sum of squares; *Φ*, fixation index.

**FIGURE 3:**
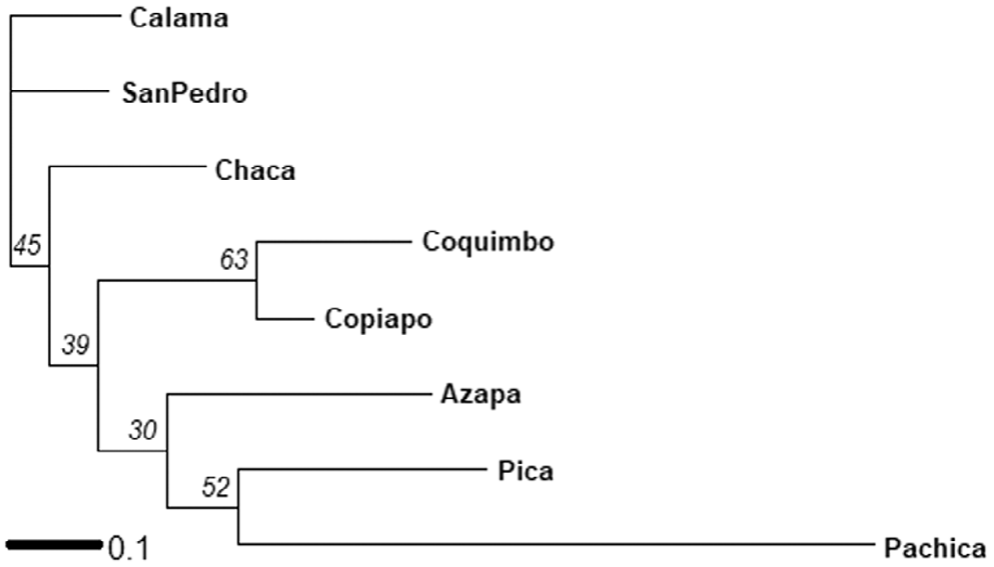
NJ representative of Nei's genetic distances between populations studied. Numbers over branches represent bootstrap support for each node.

Genetic relationships among populations were evaluated with an unrooted NJ tree based on pairwise Nei genetic distances (Figure 3). Although bootstrap support was generally low (BS < 63%), pachica is the most differentiated population, which is grouped with pica and azapa. copiapo and coquimbo formed the well‐supported group (BS = 63%).

**FIGURE 4:**
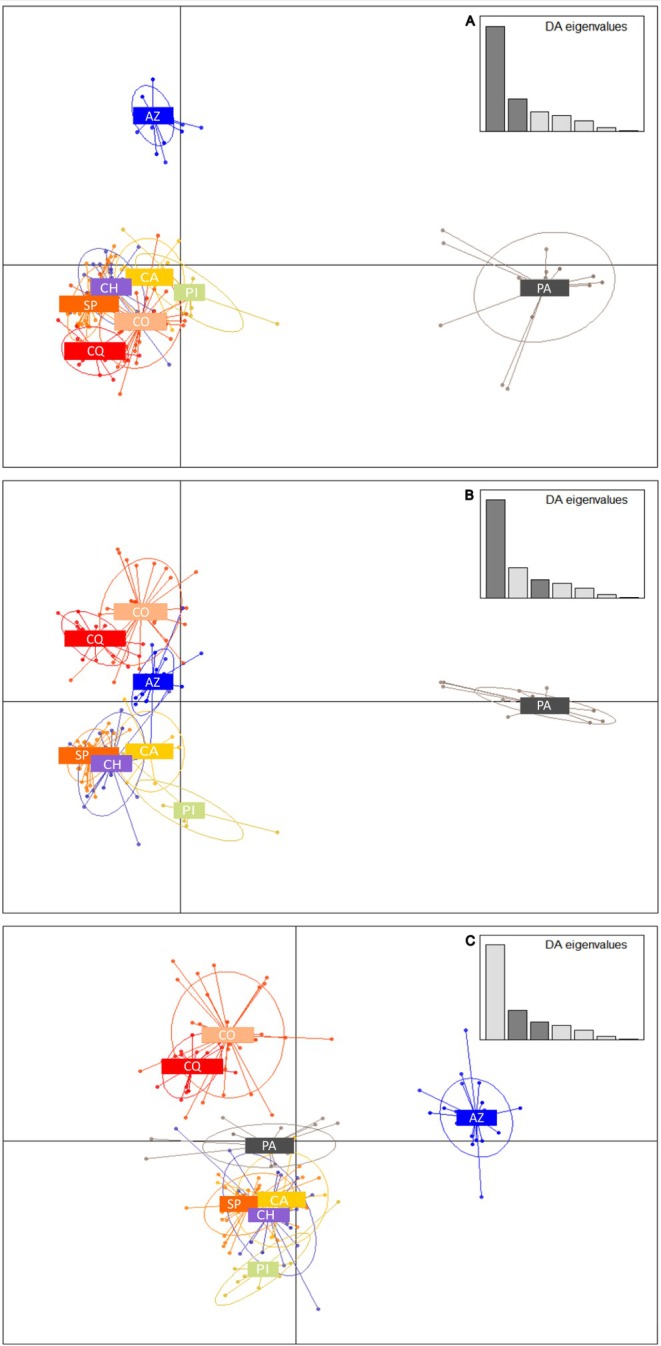
Scatterplot of individuals on the 3 principal components obtained by DAPC. (A) PC1 vs. PC2, (B) PC1 vs. PC3, (C) PC2 vs. PC3. The graph represents the individuals as dots and the groups as inertia ellipses. Eigenvalues of the analysis are displayed in inset with clusters defined a priori according to the population sampling site. AZ, Azapa; CA, Calama; CH, Chaca; CO, Copiapo; CQ, Coquimbo; PA, Pachica; PI, Pica; SP, San Pedro.

In the DAPC (Figure 4), three discriminant axes were retained, explaining 66.5% of the total variation (44.22%, 13.83% and 8.45%, respectively). Analysis of the three scatterplots revealed that not all the populations were clearly differentiated. The first two PC axes showed that pachica and azapa individuals formed distinct clear groups, whereas the individuals from the remaining populations largely overlapped. The PC1 vs. PC3 scatterplot allowed for the recognition of a group integrated by coquimbo and copiapo individuals. In the PC2 vs. PC3 scatterplot, the same pattern described above was detected while san pedro and chaca individuals overlapped extensively in all dimensions.

### 3.3 Bottlenecks and Population Size

Signals of bottleneck were evaluated and detected using three methods: (1) the heterozygosity excess test, (2) mode shift distribution, and (3) M‐ratio analysis. In the CALAMA population, all three tests showed evidence of bottleneck events, whereas in AZAPA, CHACA, COPIAPO, COQUIMBO, PACHICA, PICA, and SAN PEDRO, only two of the three tests were significant (significant cases are highlighted in gray in Table 4). In detail, the heterozygosity excess test for bottleneck events (method 1) was significant under the Infinite Allele Model (IAM) for six populations (AZAPA, CALAMA, CHACA, COPIAPO, COQUIMBO, and PACHICA), indicating departures from mutation‐drift equilibrium consistent with a recent bottleneck. Under the Two‐Phase Model (TPM), four populations (AZAPA, CALAMA, CHACA, and COQUIMBO) showed evidence of bottlenecks, whereas under the Stepwise Mutation Model (SMM), only PICA and SAN PEDRO suggested recent population size reductions. Allele frequency distributions (method 2) exhibited a mode shift in CALAMA, also consistent with a recent bottleneck. The M‐ratio test (method 3) supported historical bottleneck in all populations. The observed M‐Ratio values ranged from 0.275 (COQUIMBO) to 0.474 (PACHICA), and in all the cases were < 0.68, the threshold value considered here as indicative of ancient bottleneck occurrence.

**TABLE 4:** Heterozygosity excess^a^, mode shift distribution^b^ and M‐ratio analyses^c^ of bottleneck signature for populations of *G. decorticans* based on 9 microsatellite *loci*.

	Population	Azapa	Calama	Chaca	Copiapo	Coquimbo	Pachica	Pica	San Pedro
	Mean *N*	84.00	14.00	46.00	64.00	38.00	50.00	20.00	72.00
	Mean *K*	5.67	3.56	5.33	9.11	5.22	3.44	3.78	6.56
	Mean_*H*_E_	0.711	0.625	0.700	0.785	0.707	0.510	0.496	0.607
**Method 1 Heterozygosity excess**	**IAM**								
	Sign test *p* value	**0.007**	0.157	**0.008**	0.070	0.058	0.174	0.286	0.192
	Standarized differences test *p* value	**0.000**	**0.014**	**0.004**	**0.005**	**0.003**	**0.005**	0.447	0.191
	Wilcoxon Sign rank deficiency test *p* value (1tail)	**0.001**	**0.010**	**0.001**	**0.005**	**0.003**	**0.010**	0.371	0.213
	Wilcoxon Sign rank deficiency test *p* value (2tail)	**0.002**	**0.020**	**0.002**	**0.010**	0.006	**0.020**	0.742	0.426
	**TPM**								
	Sign test *p* value	0.060	0.432	0.201	0.469	0.065	0.206	0.189	0.540
	Standarized differences test *p* value	**0.008**	0.055	0.055	0.109	**0.037**	0.056	0.121	0.220
	Wilcoxon Sign rank deficiency test *p* value (1tail)	**0.005**	**0.014**	**0.010**	0.125	**0.019**	0.098	0.809	0.500
	Wilcoxon Sign rank deficiency test *p* value (2tail)	**0.010**	**0.027**	**0.020**	0.250	**0.037**	0.195	0.461	1.000
	**SMM**								
	Sign test *p* value	0.465	0.468	0.533	0.536	0.535	0.500	0.052	**0.027**
	Standarized differences test *p* value	0.186	0.154	0.466	0.088	0.298	0.417	**0.007**	**0.000**
	Wilcoxon Sign rank deficiency test *p* value (1tail)	0.125	0.125	0.545	0.752	0.285	0.422	0.994	0.995
	Wilcoxon Sign rank deficiency test *p* value (2tail)	0.250	0.250	1.000	0.570	0.570	0.844	**0.020**	**0.014**
**Method 2 Mode shift distribution**	**Distribution observed**	L‐shaped	**Shifted**	L‐shaped	L‐shaped	L‐shaped	L‐shaped	L‐shaped	L‐shaped
**Method 3 M‐Ratio analysis**	**M‐Ratio value (SE)**	**0.320 (0.053)**	**0.289 (0.056)**	**0.326 (0.034)**	**0.415 (0.062)**	**0.275 (0.046)**	**0.474 (0.111)**	**0.382 (0.086)**	**0.322 (0.059)**

*Note:* Values in bold and shaded mean possible signature of genetic bottleneck event. The Standardized differences and Wilcoxon Sign rank deficiency test follows Cornuet and Luikart (1997) and Luikart et al. (1998) respectively.

Abbreviations: IAM = Infinite Allele Model, Mean *N* = mean number of alleles, Mean *K* = mean number of alleles per locus, Mean *H*_E_ = expected Heterozygosity under HW, SMM = Stepwise Mutation Model, TPM = Two phase model.

^a^ Cornuet and Luikart (1997).

^b^ Luikart et al. (1998).

^c^ Garza and Williamson (2001).

The estimation of effective population size by LDNe from the nine SSR *loci* studied were low in all populations, with the highest point estimate found in COPIAPO (N_E‐LD_ = 18; Table 5). For CALAMA and PICA, the linkage disequilibrium method could not provide a finite point estimate for *N*_E_ indicating insufficient signal in the data for a reliable estimate, likely due to small sample sizes.

**TABLE 5:** Effective population size estimates obtained by the Linkage disequilibrium method of Hill (1981) (*N*_E‐LD_) implemented in NeESTIMATOR (Waples and Do 2008; Do et al. 2014) in *G. decorticans* populations.

Population	*N*_E‐LD_ [CI_95%_]
Azapa	2.4 [2.1–2.7]
Calama	Undefined [0.4–0.8]
Chaca	4.6 [2.9–7]
Copiapo	18.5 [14.7–23.7]
Coquimbo	1.8 [1.5–2.2]
Pachica	13.2 [6.5–31.7]
Pica	Undefined [0.4–0.7]
San Pedro	3.6 [3–4.6]

## 4 Discussion

This study presents the most comprehensive population‐genetic assessment to date of *G. decorticans*, a keystone woody legume endemic to the hyper‐arid Atacama Desert. By coupling nine developed microsatellite *loci* with extensive geographic sampling, we have been able to unravel how habitat mosaics of fertile valleys and barren plains shape contemporary genetic diversity, population structure, and demographic history.

### 4.1 Genetic Diversity

Across the eight sampled populations, *G. decorticans* shows a notable reservoir of genetic diversity for a tree inhabiting the world's driest non‐polar desert. Populations from the Atacama Desert showed relatively high levels of genetic diversity (mean *Ar* = 3.86, mean *H*_O_ = 0.73, mean *H*_E_ = 0.62), comparable to other woody tree species with similar life‐history traits (Hamrick and Godt 1996; Hamrick et al. 1992; Nybom 2004; Broadhurst et al. 2017). The observed heterozygosity consistently exceeded expectations in all the populations (mean *F*_IS_ = −0.192), and produced a significantly negative inbreeding coefficient in six of them. In general, heterozygote excess has several potential causes. It may result from small effective reproductive population size, overdominance, negative assortative mating, and asexual reproduction (Hamrick and Godt 1996, Keller and Waller 2002; Stoeckel et al. 2006). Additionally, recent population admixture will cause excess of heterozygotes in the offspring affecting many *loci* in a transient way (Waples et al. 2018). The pervasive and significant heterozygote excess (negative *F*_IS_) observed in several studied populations is a classic signature of recent population bottlenecks (Cornuet and Luikart 1997). Following a sudden reduction in population size, allelic diversity is eroded faster than heterozygosity, creating a transient excess of heterozygotes. This interpretation is strongly supported by our detection of recent bottleneck signals in multiple populations using heterozygosity‐excess tests and mode‐shift indicators.

Our screening for null alleles indicates that genotyping artifacts are unlikely to bias the conclusions presented here, as the null allele frequencies were generally low. Specifically, the frequency exceeded 0.10, the accepted threshold for excluding *loci* (Chapuis and Estoup 2007), in only three cases; however, this bias could not be completely ruled out. Moreover, the prevailing negative *F*_IS_ estimates obtained in the different populations contradicts the surplus of homozygotes expected if null alleles were inflating deficiency. We therefore interpreted HWE departures and LD patterns as biological signals rather than methodological artifacts. Indeed, spatial context may help explain heterogeneity in the metrics analyzed. The high genetic diversity in valleys like Azapa, Chaca, and Copiapó may be facilitated by their relatively larger and more contiguous habitat patches compared to more isolated sites, but this requires further ecological investigation. More than 1.100 km of predominantly barren landscape separates Azapa from Coquimbo, probably restricting gene flow, but the lack of isolation‐by‐distance and the high differentiation found between some geographically close populations (e.g., Pachica and Pica) suggest that the barren landscape matrix acts as a strong barrier to gene flow, overriding the effect of simple geographic distance. This dichotomy is mirrored in Hardy–Weinberg patterns: while most *loci* are at equilibrium in Chaca, widespread heterozygote deficits occur in Pachica and Coquimbo, consistent with Wahlund effects or recent contractions (Contreras et al. 2021).

In population genetic studies, estimates of genetic diversity, differentiation, and structure should ideally be based on physically unlinked *loci*. Linkage disequilibrium (LD) test here performed suggests that the forces causing LD are acting differently in each population and are consistent with no physically linked *loci*. The linkage disequilibrium observed between *loci* can be caused by numerous factors including, but not limited to, physical proximity. Other factors that could have contributed are population differentiation, asexual reproduction, and natural selection (Agapow and Burt 2001). LD may imply reduced recombination owing to chronically small effective sizes or founder events according to Luikart and Cornuet (1998). Taken together, the high diversity across populations, the generally low null allele incidence, the heterozygote excess, and the population‐specific yet widespread LD depict historically large, well‐recombined populations that have recently fragmented into valley refugia, where reduced Ne, drift, and inbreeding increasingly shape allele‐frequency dynamics. The genetic structure here detected and/or demographic history is in agreement with the LD detected.

### 4.2 Population Differentiation and Structure

The global estimate of genetic differentiation (*F*_ST_ = 0.208; *p* < 0.001) places *G. decortican*s in the upper tier of outcrossing trees with fragmented ranges, rivaling values reported for Mediterranean pines and African acacias (Hamrick and Godt 1996; Nybom 2004; Broadhurst et al. 2017). Hierarchical analyses confirm a spatially nested structure. AMOVA attributes 89.8% of genetic variance to within‐individual differences and only 10.2% to among‐population divergence (ΦST = 0.102). Such partitioning is classical for wind‐ or animal‐pollinated woody perennials (Excoffier et al. 1992). Nevertheless, our Mantel test rejects a simple isolation‐by‐distance scenario, implying that gene flow patterns could be attributed to other causes. In this sense, isolation may be better predicted by habitat quality, groundwater, fog drip, and vegetated corridors than by Euclidean spacing (Wang and Bradburd 2014).

Topology from the Nei‐based neighbor‐joining tree and scatterplots based on a discriminant analysis of principal components (DAPC) are congruent. Both methods separate a northern cluster (Pachica, Azapa) from a southern coastal clade (Copiapó, Coquimbo), while San Pedro and Chaca occupy an intermediate, partly overlapping position. The low differentiation between some populations, such as San Pedro and Chaca, could be maintained by ongoing pollen flow, possibly via generalist pollinators capable of moving long distances across the desert matrix. Direct observations of pollinator movements are needed to test this hypothesis.

Altogether, *G. decorticans* exhibits a hierarchically complex genetic mosaic. Our results suggest that valleys are acting as semi‐permeable conduits, whereas intervening desert plains seem to function as formidable barriers for *G. decorticans* . This interplay generates both sharp discontinuities and diffuse zones of low population differentiation, emphasizing that conservation planning should account for environmental corridors rather than rely solely on geographic proximity.

### 4.3 Bottlenecks and Population Size

Three complementary approaches reveal that demographic contractions are widespread and recurrent in *G. decorticans* populations. For recent demography events, we first analyze heterozygosity‐excess tests under the Infinite Allele Model (IAM) and detected significant bottleneck signals in Azapa, Calama, Chaca, Copiapó, Coquimbo, and Pachica. Because allelic richness erodes faster than heterozygosity following a sudden size reduction, an excess of heterozygotes is a reliable sign of recent decline (Cornuet and Luikart 1997). A shifted allele‐frequency distribution (Luikart et al. 1998) found in Calama corroborates this inference. Under the Stepwise Mutation Model (SMM), only Pica and San Pedro retain significance, but this discrepancy is to be expected when bottlenecks occurred < 100 generations ago (Peery et al. 2012). While it is appropriate to emphasize the TPM and SMM results as they best represent SSR mutation patterns (Piry et al. 1999), the overall evidence consistently points to recent bottlenecks as a widespread phenomenon in *G. decorticans* populations. In addition, assuming typical microsatellite mutation parameters, the universally low M‐ratios (all < 0.68) are strongly indicative of historical, prolonged reductions in population size (Garza and Williamson 2001). Considering that M‐ratio integrates over longer time horizons than heterozygosity‐excess tests, the concurrence of both metrics may imply a pattern of recurrent bottleneck events rather than a single synchronous episode. A limitation should be acknowledged, as bottleneck tests based on allele frequencies generally assume random mating and the absence of population substructure; thus, the detected HWE deviations may represent a potential bias.

Finally, our estimates of contemporary effective population size are disquietingly low and punctual linkage‐disequilibrium *N*e values do not exceed 18. Limitations of this estimation method should be considered as it assumes that markers segregate independently, and the precision of the estimate is highly sensitive to allelic sampling error (which results in wide confidence intervals), particularly when the sample size is reduced. Here, for the populations where Ne could be estimated (Table 5), all values are well below the general conservation guidelines of Ne > 50 to mitigate short‐term inbreeding depression and Ne > 500 for long‐term adaptive potential (Frankham et al. 2014). Such small *N*e, combined with significant LD, points to non‐equilibrium demography and strong genetic drift. In the Atacama, demographic contractions are plausibly triggered by multi‐year droughts, groundwater depletion, and livestock browsing, each capable of truncating census size within a few generations (Decuyper et al. 2016; Xiao and Huang 2016; Garreaud et al. 2020; Patón 2021). Because dispersal of seeds is largely restricted to short distances by gravity, and pollen movement depends on pollinators whose activity declines under resource scarcity, even demographic recovery may not rebuild pre‐bottleneck diversity. Given *G. decorticans* 'capacity for clonal persistence and its relatively long generation times, the resulting slow genetic turnover leaves lasting genetic signatures of collapse that may persist across multiple generations.

In sum, *G. decorticans* populations are genetically diverse yet demographically fragile: high heterozygosity coexists with widespread LD and critically low *N*e, reflecting historical connectivity punctuated by repeated size collapses. Any future management must therefore confront the dual challenge of sustaining valley corridors that enable gene flow while bolstering effective sizes to buffer against further drift effects.

### 4.4 Conservation Implications

Appropriate management of native species depends on mapping their nature variation and population structure throughout their entire distribution range (Mattera et al. 2020). The genetic context described in the present paper carries important conservation aspects to be considered. Priority populations may be based on a clear combination of the genetic data here obtained considering the low diversity found in some populations like Pachica and Pica, the strong bottleneck signals described in all the populations and mainly in Calama, and the high uniqueness described for Pachica individuals.

Assisted population migration strategy (using materials from genetically rich sources as seeds/propagules) requires a careful balance between contrasting risks. On one hand, it entails the risk of producing outbreeding depression due to the generation of maladapted hybrids originated by inter‐population crossing adapted to different local conditions. On the other hand, inbreeding depression may also occur due to an increase of self‐pollination or breeding between related parents if assortative mating occurs between individuals from the same source (McKay et al. 2005; Breed et al. 2013; Thomas et al. 2014). In this sense, our first recommendation before suggesting the movement of material across sites is the installation of crossing provenance trials to fully evaluate its consequences.

The assisted gene flow from nearby, genetically similar, but more diverse populations rather than from the most diverse populations from distant sources can be considered. For this purpose, a deep comprehensive characterization of the *G. decorticans* population structure is essential and valuable for delineating future particular restoration plans. This allows for the informed selection of seeds and/or propagules (whether local or non‐local) from the same and/or different areas. In each case, the potential benefits versus risks must always be judiciously evaluated and kept in mind.

Finally, preserving valley corridors is crucial as these habitats may function as refugia and stepping‐stones sustaining the limited connectivity that remains among populations (Krosby et al. 2018; Szcodronski et al. 2024). In this sense, genetic monitoring performed periodically will be vital because genetic recovery often lags behind census size increases (Pierson et al. 2016). Given the low Ne and bottleneck signals, establishing a program to monitor genetic parameters over time is critical for detecting further erosion and evaluating the success of conservation interventions. Integrating habitat‐suitability modeling with genetic data will further refine strategies for maintaining adaptive potential in a desert projected to become even drier (Fitzpatrick and Keller 2015; Garreaud et al. 2020).

In conclusion, this study presents the most comprehensive genetic evaluation of *G. decorticans* to date, revealing moderate to high levels of genetic diversity and significant differentiation among populations. The signals of genetic bottlenecks detected in several populations and the reduced effective population sizes estimates are likely driven by habitat fragmentation, limited gene flow, and environmental constraints typical of the Atacama Desert. Based on these results, conservation strategies should prioritize populations based on genetic information including those with relatively low diversity, strong bottleneck signals, and high uniqueness; promote careful evaluation of source populations before implementing assisted gene flow programs; and protect habitat corridors to maintain connectivity. Long‐term monitoring of genetic parameters will be critical to detect ongoing changes and ensure the persistence of this ecologically important desert tree in the face of increasing climatic and anthropogenic pressures.

## Author Contributions

**Roberto Contreras:** conceptualization (equal), data curation (equal), funding acquisition (lead), investigation (equal), methodology (equal), supervision (equal), visualization (equal), writing – original draft (equal), writing – review and editing (equal). **Mariana Arias‐Aburto:** data curation (equal), investigation (supporting). **Cecilia Bessega:** conceptualization (equal), formal analysis (lead), investigation (equal), methodology (equal), supervision (equal), visualization (equal), writing – original draft (equal), writing – review and editing (lead).

## Funding

This work was supported by ANID—MILENIO, NCS2022_024, DIUDA ‐ UNIVERSIDAD DE ATACAMA, 22423, and ANID‐FONDECYT, 11230668.

## Conflicts of Interest

The authors declare no conflicts of interest.

## Supporting information


**Table S1:** Nine SSR *loci* of *Geoffroea decorticans* used in the present study. The raw sequencing data used to develop the SSR markers for *G. decorticans* are publicly available in the NCBI Sequence Read Archive (SRA) under BioProject ID PRJNA719569 and BioSample ID SAMN18613292. The first six *loci* listed were previously used in Contreras et al. (2021).
**Table S2:** Linkage disequilibrium for each of the microsatellite *loci* pairs within *G. decorticans* sampled populations obtained by Markov Chain Monte Carlo (MCMC) algorithm implemented in genepop (Rousset 2008). Pairs of *loci* with deviations from linkage equilibrium after FDR correction (Benjamini and Hochberg 1995) are highlighted. *p* val: the estimated probability of genotype independence, SE: standard error of the *p*‐value estimate, Switches: the total number of successful state changes made by the Markov chain, *p* adj: corrected *p* value considering FDR correction.
**Table S3:** H‐W equilibrium test for each *loci* in the eight populations of *G. decorticans* .

## Data Availability

Sampling locations are detailed in Figure 1 and raw data set is available at: DOI: https://doi.org/10.5061/dryad.9zw3r22wf.
