# Supplementary material for: Genetic Diversity, Population Structure and Evidence of Genetic Bottleneck in Geoffroea decorticans (Fabaceae): Implications for Conservation
Source: Ecol Evol. 2026 Apr 29;16(5):e73527. doi: 10.1002/ece3.73527 (PMC13125962; doi:10.1002/ece3.73527)
Supplement: Supplementary file 1 — Table S1: Nine SSR loci of Geoffroea decorticans used in the present study. The raw sequencing data used to develop the SSR markers for G. decorticans are publicly available in the NCBI Sequence Read Archive (SRA) under BioProject ID PRJNA719569 and BioSample ID SAMN18613292. The first six loci listed were previously used in Contreras et al. (2021). Table S2: Linkage disequilibrium for each of the microsatellite loci pairs within G. decorticans sampled populations obtained by Markov Chain Monte Carlo (MCMC) algorithm implemented in genepop (Rousset 2008). Pairs of loci with deviations from linkage equilibrium after FDR correction (Benjamini and Hochberg 1995) are highlighted. p val: the estimated probability of genotype independence, SE: standard error of the p‐value estimate, Switches: the total number of successful state changes made by the Markov chain, p adj: corrected p value considering FDR correction. Table S3: H‐W equilibrium test for each loci in the eight populations of G. decorticans . [file ECE3-16-e73527-s001.docx]

**SUPPORTING INFORMATION**

**Table S1.** Nine SSR *loci* of *Geoffroea decorticans* used in the present study. The raw sequencing data used to develop the SSR markers for for *G. decorticans* are publicly available in the NCBI Sequence Read Archive (SRA) under BioProject ID PRJNA719569 and BioSample ID SAMN18613292. The first six *loci* listed were previously used in Contreras et al. (2021).

| **Locus name** | **Motif** | **Primer sequence (5’–3’)** | **Product size (bp)** | **Annealing temp.(°C)** |
| --- | --- | --- | --- | --- |
| SSRGD27558 | (GT)_14_ | F: GAGATGAAGCTTACGCTGGAA  R: TTGAACGAACTGGAAACTATGG | 150 bp | 58 °C |
| SSRGD11258 | (CT)_26_ | F: GTGAGCGTTGACCCTTCC  R AGATTCAGAGAAGTTGCAAAGG | 136 bp | 60 °C |
| SSRGD7345 | (ATC)_13_ | F: ACCATGATCACAACCACCAA  R: ATACAAGTGGAACCAACATTTAGATA | 120 bp | 55 °C |
| SSRGD17951 | (AG)_24_ | F: CAAATTGGGAGCGAGCTAAG  R: CTTGTTGTTGTTGCCACTGC | 186 bp | 59 °C |
| SSRGD11733 | (CT)_21_ | F: GGGTTTCAAAGTTCATTTACCAA  R: AAGCAGAACCCACATGCTCT | 113 bp | 55 °C |
| SSRGD17837 | (AG)_28_ | F: GCGGTGGGTTATAGTGGAGA  R: TCCCTCACTAGCTCCTCTTCC | 147 bp | 59 °C |
| SSRGD8699 | (TAA)_11_ | F: CCACGCACAAAAGGTTAAGC  R: TCCCAACCATACCAACTTTCA | 138 bp | 55 °C |
| SSRGD8997 | (GTT)_12_ATTG(TTA)_3_ | F: TGGATGGTATTATTTGGCATGT  R: TCTTTCGTGAGTTTTAATGGATTT | 225 bp | 55 °C |
| SSRGD13517 | (TTA)_14_T(CAT)_3_ | F: ACTTTGGTTACTATAGTTGGGGA  R: TGCATTGGATGTAGGGTAAATGT | 150 bp | 58 °C |

**Table S2.** Linkage disequilibrium for each of the microsatellite *loci* pairs within *G. decorticans* sampled populations obtained by Markov Chain Monte Carlo (MCMC) algorithm implemented in genepop (Rousset, 2008). Pairs of *loci* with deviations from linkage equilibrium after FDR correction (Benjamini & Hochberg, 1995) are highlighted. P val: the estimated probability of genotype independence, SE: standard error of the P-Value estimate, Switches: the total number of successful state changes made by the Markov chain, P adj: corrected P value considering FDR correction.

|  |  |  | Azapa |  |  |  | Calama |  |  |
| --- | --- | --- | --- | --- | --- | --- | --- | --- | --- |
| Locus 1 | Locus 2 | P val | SE | Switches | P adj | P val | SE | Switches | P adj |
| SSRGD11258 | SSRGD11733 | 0.000 | 0.000 | 80283 | 0.000 | 0.198 | 0.002 | 103602 | 0.227 |
| SSRGD11258 | SSRGD13517 | 0.000 | 0.000 | 66862 | 0.000 | 0.097 | 0.001 | 110805 | 0.116 |
| SSRGD11258 | SSRGD17837 | 0.000 | 0.000 | 55647 | 0.000 | 0.095 | 0.001 | 190281 | 0.116 |
| SSRGD11258 | SSRGD17951 | 0.000 | 0.000 | 66267 | 0.000 | 0.096 | 0.001 | 190500 | 0.116 |
| SSRGD11258 | SSRGD7345 | 0.634 | 0.003 | 114872 | 0.666 | 0.096 | 0.001 | 190718 | 0.116 |
| SSRGD11258 | SSRGD8699 | 0.000 | 0.000 | 98655 | 0.000 | 0.096 | 0.001 | 190531 | 0.116 |
| SSRGD11258 | SSRGD8997 | 0.000 | 0.000 | 65737 | 0.000 | 0.200 | 0.002 | 103442 | 0.227 |
| SSRGD11733 | SSRGD13517 | 0.000 | 0.000 | 60077 | 0.000 | 1.000 | 0.000 | 65454 | 1.000 |
| SSRGD11733 | SSRGD17837 | 0.000 | 0.000 | 50396 | 0.000 | 0.716 | 0.002 | 139311 | 0.733 |
| SSRGD11733 | SSRGD8699 | 0.000 | 0.000 | 98700 | 0.000 | 0.715 | 0.002 | 138517 | 0.733 |
| SSRGD11733 | SSRGD8997 | 0.000 | 0.000 | 62196 | 0.000 | 1.000 | 0.000 | 64415 | 1.000 |
| SSRGD17837 | SSRGD13517 | 0.000 | 0.000 | 38628 | 0.000 | 0.048 | 0.001 | 134497 | 0.064 |
| SSRGD17951 | SSRGD11733 | 0.000 | 0.000 | 63318 | 0.000 | 0.713 | 0.002 | 138945 | 0.733 |
| SSRGD17951 | SSRGD13517 | 0.000 | 0.000 | 52453 | 0.000 | 0.049 | 0.001 | 135470 | 0.064 |
| SSRGD17951 | SSRGD17837 | 0.000 | 0.000 | 43626 | 0.000 | 0.047 | 0.001 | 261663 | 0.064 |
| SSRGD17951 | SSRGD8699 | 0.000 | 0.000 | 80738 | 0.000 | 0.048 | 0.001 | 262166 | 0.064 |
| SSRGD17951 | SSRGD8997 | 0.000 | 0.000 | 52181 | 0.000 | 0.094 | 0.001 | 139016 | 0.116 |
| SSRGD27558 | SSRGD11258 | 0.000 | 0.000 | 63564 | 0.000 | 0.095 | 0.001 | 190148 | 0.116 |
| SSRGD27558 | SSRGD11733 | 0.000 | 0.000 | 58641 | 0.000 | 0.715 | 0.002 | 138457 | 0.733 |
| SSRGD27558 | SSRGD13517 | 0.000 | 0.000 | 45037 | 0.000 | 0.048 | 0.001 | 135043 | 0.064 |
| SSRGD27558 | SSRGD17837 | 0.000 | 0.000 | 38396 | 0.000 | 0.048 | 0.000 | 261743 | 0.064 |
| SSRGD27558 | SSRGD17951 | 0.000 | 0.000 | 50241 | 0.000 | 0.047 | 0.001 | 262137 | 0.064 |
| SSRGD27558 | SSRGD7345 | 0.000 | 0.000 | 96167 | 0.000 | 0.048 | 0.000 | 262415 | 0.064 |
| SSRGD27558 | SSRGD8699 | 0.000 | 0.000 | 79699 | 0.000 | 0.048 | 0.001 | 262446 | 0.064 |
| SSRGD27558 | SSRGD8997 | 0.000 | 0.000 | 47076 | 0.000 | 0.096 | 0.001 | 138901 | 0.116 |
| SSRGD7345 | SSRGD11733 | 0.000 | 0.000 | 119709 | 0.000 | 0.716 | 0.001 | 139245 | 0.733 |
| SSRGD7345 | SSRGD13517 | 0.000 | 0.000 | 105836 | 0.000 | 0.047 | 0.001 | 134461 | 0.064 |
| SSRGD7345 | SSRGD17837 | 0.000 | 0.000 | 86508 | 0.000 | 0.047 | 0.000 | 262085 | 0.064 |
| SSRGD7345 | SSRGD17951 | 0.096 | 0.002 | 94397 | 0.116 | 0.048 | 0.001 | 261806 | 0.064 |
| SSRGD7345 | SSRGD8699 | 0.000 | 0.000 | 137255 | 0.000 | 0.048 | 0.001 | 262657 | 0.064 |
| SSRGD7345 | SSRGD8997 | 0.000 | 0.000 | 99050 | 0.000 | 0.095 | 0.001 | 138086 | 0.116 |
| SSRGD8699 | SSRGD13517 | 0.000 | 0.000 | 84773 | 0.000 | 0.047 | 0.001 | 134898 | 0.064 |
| SSRGD8699 | SSRGD17837 | 0.000 | 0.000 | 70441 | 0.000 | 0.048 | 0.001 | 262521 | 0.064 |
| SSRGD8699 | SSRGD8997 | 0.000 | 0.000 | 82230 | 0.000 | 0.096 | 0.001 | 139122 | 0.116 |
| SSRGD8997 | SSRGD13517 | 0.000 | 0.000 | 47843 | 0.000 | 0.048 | 0.001 | 65086 | 0.064 |
| SSRGD8997 | SSRGD17837 | 0.000 | 0.000 | 40867 | 0.000 | 0.095 | 0.001 | 138760 | 0.116 |
|  |  |  | Chaca |  |  |  |  | Copiapo |  |
| SSRGD11258 | SSRGD11733 | 0.024 | 0.004 | 20086 | 0.037 | 0.011 | 0.006 | 5433 | 0.020 |
| SSRGD11258 | SSRGD13517 | 0.000 | 0.000 | 31904 | 0.000 | 0.015 | 0.004 | 13250 | 0.026 |
| SSRGD11258 | SSRGD17837 | 0.018 | 0.002 | 53903 | 0.031 | 0.000 | 0.000 | 2876 | 0.000 |
| SSRGD11258 | SSRGD17951 | 0.014 | 0.002 | 32798 | 0.025 | 0.001 | 0.000 | 9162 | 0.002 |
| SSRGD11258 | SSRGD7345 | 0.007 | 0.002 | 26424 | 0.013 | 0.000 | 0.000 | 19695 | 0.001 |
| SSRGD11258 | SSRGD8699 | 0.000 | 0.000 | 22972 | 0.001 | 0.000 | 0.000 | 5544 | 0.000 |
| SSRGD11258 | SSRGD8997 | 0.134 | 0.005 | 39382 | 0.157 | 0.000 | 0.000 | 4823 | 0.000 |
| SSRGD11733 | SSRGD13517 | 0.000 | 0.000 | 19339 | 0.000 | 0.054 | 0.007 | 16477 | 0.071 |
| SSRGD11733 | SSRGD17837 | 0.000 | 0.000 | 38957 | 0.000 | 0.000 | 0.000 | 4649 | 0.000 |
| SSRGD11733 | SSRGD8699 | 0.000 | 0.000 | 13366 | 0.000 | 0.000 | 0.000 | 8276 | 0.000 |
| SSRGD11733 | SSRGD8997 | 0.001 | 0.000 | 27161 | 0.002 | 0.003 | 0.002 | 7205 | 0.007 |
| SSRGD17837 | SSRGD13517 | 0.000 | 0.000 | 58409 | 0.000 | 0.001 | 0.000 | 11638 | 0.002 |
| SSRGD17951 | SSRGD11733 | 0.003 | 0.001 | 21421 | 0.007 | 0.002 | 0.001 | 12005 | 0.003 |
| SSRGD17951 | SSRGD13517 | 0.000 | 0.000 | 34248 | 0.001 | 0.105 | 0.009 | 23396 | 0.125 |
| SSRGD17951 | SSRGD17837 | 0.000 | 0.000 | 57649 | 0.000 | 0.000 | 0.000 | 8082 | 0.000 |
| SSRGD17951 | SSRGD8699 | 0.000 | 0.000 | 24526 | 0.001 | 0.000 | 0.000 | 13394 | 0.000 |
| SSRGD17951 | SSRGD8997 | 0.211 | 0.006 | 42226 | 0.235 | 0.000 | 0.000 | 11877 | 0.001 |
| SSRGD27558 | SSRGD11258 | 0.108 | 0.004 | 66526 | 0.128 | 0.005 | 0.004 | 3966 | 0.010 |
| SSRGD27558 | SSRGD11733 | 0.022 | 0.002 | 49930 | 0.036 | 0.000 | 0.000 | 6222 | 0.000 |
| SSRGD27558 | SSRGD13517 | 0.000 | 0.000 | 72405 | 0.000 | 0.005 | 0.002 | 14938 | 0.011 |
| SSRGD27558 | SSRGD17837 | 0.002 | 0.000 | 106119 | 0.004 | 0.000 | 0.000 | 3422 | 0.000 |
| SSRGD27558 | SSRGD17951 | 0.002 | 0.000 | 70651 | 0.004 | 0.001 | 0.000 | 10394 | 0.002 |
| SSRGD27558 | SSRGD7345 | 0.000 | 0.000 | 58456 | 0.000 | 0.024 | 0.004 | 22475 | 0.038 |
| SSRGD27558 | SSRGD8699 | 0.087 | 0.004 | 53910 | 0.110 | 0.000 | 0.000 | 6165 | 0.000 |
| SSRGD27558 | SSRGD8997 | 0.040 | 0.002 | 81468 | 0.058 | 0.001 | 0.001 | 5516 | 0.001 |
| SSRGD7345 | SSRGD11733 | 0.000 | 0.000 | 16954 | 0.000 | 0.019 | 0.003 | 25101 | 0.033 |
| SSRGD7345 | SSRGD13517 | 0.000 | 0.000 | 27844 | 0.000 | 0.479 | 0.008 | 45959 | 0.520 |
| SSRGD7345 | SSRGD17837 | 0.000 | 0.000 | 47643 | 0.000 | 0.000 | 0.000 | 17090 | 0.000 |
| SSRGD7345 | SSRGD17951 | 0.000 | 0.000 | 27951 | 0.000 | 0.002 | 0.001 | 35625 | 0.004 |
| SSRGD7345 | SSRGD8699 | 0.000 | 0.000 | 19961 | 0.000 | 0.000 | 0.000 | 28774 | 0.000 |
| SSRGD7345 | SSRGD8997 | 0.000 | 0.000 | 34107 | 0.000 | 0.009 | 0.002 | 25241 | 0.016 |
| SSRGD8699 | SSRGD13517 | 0.000 | 0.000 | 23197 | 0.000 | 0.002 | 0.001 | 18993 | 0.004 |
| SSRGD8699 | SSRGD17837 | 0.000 | 0.000 | 42906 | 0.000 | 0.000 | 0.000 | 4613 | 0.000 |
| SSRGD8699 | SSRGD8997 | 0.013 | 0.002 | 30399 | 0.023 | 0.000 | 0.000 | 7500 | 0.000 |
| SSRGD8997 | SSRGD13517 | 0.003 | 0.001 | 41944 | 0.007 | 0.010 | 0.003 | 16455 | 0.018 |
| SSRGD8997 | SSRGD17837 | 0.007 | 0.001 | 67825 | 0.013 | 0.000 | 0.000 | 4079 | 0.000 |
|  |  |  | Coquimbo |  |  |  | Pachica |  |  |
| SSRGD11258 | SSRGD11733 | 0.000 | 0.000 | 20057 | 0.000 | 0.688 | 0.005 | 47640 | 0.719 |
| SSRGD11258 | SSRGD13517 | 0.000 | 0.000 | 63209 | 0.000 | 0.001 | 0.000 | 52824 | 0.002 |
| SSRGD11258 | SSRGD17837 | 0.000 | 0.000 | 41757 | 0.000 | 0.029 | 0.002 | 31895 | 0.044 |
| SSRGD11258 | SSRGD17951 | 0.026 | 0.001 | 73380 | 0.040 |  | No contingency table | |  |
| SSRGD11258 | SSRGD7345 | 0.001 | 0.000 | 140143 | 0.002 |  | No contingency table | |  |
| SSRGD11258 | SSRGD8699 | 0.000 | 0.000 | 54692 | 0.000 |  | No contingency table | |  |
| SSRGD11258 | SSRGD8997 | 0.003 | 0.001 | 39591 | 0.007 | 0.041 | 0.003 | 32035 | 0.059 |
| SSRGD11733 | SSRGD13517 | 0.002 | 0.000 | 51808 | 0.004 | 0.217 | 0.004 | 66557 | 0.241 |
| SSRGD11733 | SSRGD17837 | 0.001 | 0.001 | 32016 | 0.003 | 1.000 | 0.000 | 36847 | 1.000 |
| SSRGD11733 | SSRGD8699 | 0.000 | 0.000 | 42985 | 0.000 |  |  |  |  |
| SSRGD11733 | SSRGD8997 | 0.001 | 0.001 | 30770 | 0.003 | 0.005 | 0.001 | 38326 | 0.010 |
| SSRGD17837 | SSRGD13517 | 0.000 | 0.000 | 90311 | 0.000 | 0.009 | 0.001 | 41562 | 0.017 |
| SSRGD17951 | SSRGD11733 | 0.029 | 0.002 | 59084 | 0.045 |  | No contingency table | |  |
| SSRGD17951 | SSRGD13517 | 0.004 | 0.000 | 138731 | 0.008 |  | No contingency table | |  |
| SSRGD17951 | SSRGD17837 | 0.001 | 0.000 | 103569 | 0.002 |  | No contingency table | |  |
| SSRGD17951 | SSRGD8699 | 0.005 | 0.000 | 123799 | 0.010 |  | No contingency table | |  |
| SSRGD17951 | SSRGD8997 | 0.040 | 0.002 | 96324 | 0.058 |  | No contingency table | |  |
| SSRGD27558 | SSRGD11258 | 0.000 | 0.000 | 42827 | 0.000 |  | No contingency table | |  |
| SSRGD27558 | SSRGD11733 | 0.003 | 0.001 | 33973 | 0.007 |  | No contingency table | |  |
| SSRGD27558 | SSRGD13517 | 0.001 | 0.000 | 84073 | 0.001 |  | No contingency table | |  |
| SSRGD27558 | SSRGD17837 | 0.000 | 0.000 | 60863 | 0.001 |  | No contingency table | |  |
| SSRGD27558 | SSRGD17951 | 0.201 | 0.003 | 95620 | 0.227 |  | No contingency table | |  |
| SSRGD27558 | SSRGD7345 | 0.077 | 0.001 | 159453 | 0.099 |  | No contingency table | |  |
| SSRGD27558 | SSRGD8699 | 0.000 | 0.000 | 75039 | 0.000 |  | No contingency table | |  |
| SSRGD27558 | SSRGD8997 | 0.000 | 0.000 | 57253 | 0.000 |  | No contingency table | |  |
| SSRGD7345 | SSRGD11733 | 0.037 | 0.001 | 125617 | 0.055 |  | No contingency table | |  |
| SSRGD7345 | SSRGD13517 | 0.079 | 0.001 | 212851 | 0.101 |  | No contingency table | |  |
| SSRGD7345 | SSRGD17837 | 0.006 | 0.000 | 174579 | 0.012 |  | No contingency table | |  |
| SSRGD7345 | SSRGD17951 | 0.001 | 0.000 | 232133 | 0.001 |  | No contingency table | |  |
| SSRGD7345 | SSRGD8699 | 0.000 | 0.000 | 193440 | 0.001 |  | No contingency table | |  |
| SSRGD7345 | SSRGD8997 | 0.320 | 0.003 | 163571 | 0.352 |  | No contingency table | |  |
| SSRGD8699 | SSRGD13517 | 0.000 | 0.000 | 109749 | 0.000 |  | No contingency table | |  |
| SSRGD8699 | SSRGD17837 | 0.000 | 0.000 | 79523 | 0.000 |  | No contingency table | |  |
| SSRGD8699 | SSRGD8997 | 0.000 | 0.000 | 74633 | 0.000 |  | No contingency table | |  |
| SSRGD8997 | SSRGD13517 | 0.030 | 0.001 | 84053 | 0.045 | 0.468 | 0.006 | 42756 | 0.510 |
| SSRGD8997 | SSRGD17837 | 0.000 | 0.000 | 59212 | 0.001 | 0.309 | 0.007 | 22847 | 0.341 |
|  |  |  | Pica |  |  |  | San Pedro |  |  |
| SSRGD11258 | SSRGD11733 | 0.626 | 0.006 | 32798 | 0.663 | 0.985 | 0.005 | 6466 | 0.997 |
| SSRGD11258 | SSRGD13517 | 0.800 | 0.003 | 50036 | 0.813 | 0.000 | 0.000 | 19745 | 0.000 |
| SSRGD11258 | SSRGD17837 | 0.626 | 0.006 | 32906 | 0.663 | 0.000 | 0.000 | 22430 | 0.000 |
| SSRGD11258 | SSRGD17951 | 0.628 | 0.006 | 32606 | 0.663 | 0.000 | 0.000 | 19616 | 0.000 |
| SSRGD11258 | SSRGD7345 |  | No contingency table | |  | 0.005 | 0.001 | 45353 | 0.009 |
| SSRGD11258 | SSRGD8699 | 0.623 | 0.006 | 32595 | 0.663 | 0.511 | 0.018 | 17400 | 0.552 |
| SSRGD11258 | SSRGD8997 | 0.629 | 0.006 | 32660 | 0.663 | 0.009 | 0.002 | 23564 | 0.017 |
| SSRGD11733 | SSRGD13517 | 0.201 | 0.002 | 50412 | 0.227 | 0.034 | 0.006 | 15707 | 0.051 |
| SSRGD11733 | SSRGD17837 | 0.024 | 0.001 | 42715 | 0.037 | 0.054 | 0.007 | 16852 | 0.071 |
| SSRGD11733 | SSRGD8699 | 0.023 | 0.001 | 42863 | 0.037 | 0.000 | 0.000 | 12361 | 0.000 |
| SSRGD11733 | SSRGD8997 | 0.022 | 0.001 | 41916 | 0.036 | 0.025 | 0.004 | 17535 | 0.039 |
| SSRGD17837 | SSRGD13517 | 0.202 | 0.002 | 50325 | 0.227 | 0.000 | 0.000 | 33712 | 0.000 |
| SSRGD17951 | SSRGD11733 | 0.021 | 0.001 | 42325 | 0.036 | 0.010 | 0.003 | 14796 | 0.019 |
| SSRGD17951 | SSRGD13517 | 0.202 | 0.002 | 50136 | 0.227 | 0.000 | 0.000 | 30272 | 0.000 |
| SSRGD17951 | SSRGD17837 | 0.022 | 0.001 | 42357 | 0.036 | 0.000 | 0.000 | 35834 | 0.000 |
| SSRGD17951 | SSRGD8699 | 0.022 | 0.001 | 42478 | 0.036 | 0.042 | 0.004 | 31232 | 0.061 |
| SSRGD17951 | SSRGD8997 | 0.022 | 0.001 | 42536 | 0.036 | 0.000 | 0.000 | 38785 | 0.000 |
| SSRGD27558 | SSRGD11258 | 0.622 | 0.006 | 32692 | 0.663 | 0.097 | 0.008 | 14477 | 0.116 |
| SSRGD27558 | SSRGD11733 | 0.023 | 0.001 | 42990 | 0.036 | 0.164 | 0.009 | 11817 | 0.191 |
| SSRGD27558 | SSRGD13517 | 0.199 | 0.002 | 49744 | 0.227 | 0.000 | 0.000 | 21523 | 0.000 |
| SSRGD27558 | SSRGD17837 | 0.024 | 0.001 | 43107 | 0.037 | 0.029 | 0.002 | 26320 | 0.045 |
| SSRGD27558 | SSRGD17951 | 0.023 | 0.001 | 42469 | 0.036 | 0.000 | 0.000 | 22380 | 0.000 |
| SSRGD27558 | SSRGD7345 |  |  |  |  | 0.190 | 0.004 | 46878 | 0.222 |
| SSRGD27558 | SSRGD8699 | 0.022 | 0.001 | 42492 | 0.036 | 0.734 | 0.007 | 21988 | 0.748 |
| SSRGD27558 | SSRGD8997 | 0.023 | 0.001 | 42358 | 0.036 | 0.086 | 0.003 | 27082 | 0.110 |
| SSRGD7345 | SSRGD11733 |  | No contingency table | |  | 0.065 | 0.005 | 37274 | 0.084 |
| SSRGD7345 | SSRGD13517 |  | No contingency table | |  | 0.000 | 0.000 | 58561 | 0.000 |
| SSRGD7345 | SSRGD17837 |  | No contingency table | |  | 0.000 | 0.000 | 68894 | 0.000 |
| SSRGD7345 | SSRGD17951 |  | No contingency table | |  | 0.000 | 0.000 | 65201 | 0.000 |
| SSRGD7345 | SSRGD8699 |  | No contingency table | |  | 0.412 | 0.006 | 65356 | 0.452 |
| SSRGD7345 | SSRGD8997 |  | No contingency table | |  | 0.000 | 0.000 | 75714 | 0.000 |
| SSRGD8699 | SSRGD13517 | 0.204 | 0.002 | 50644 | 0.228 | 0.030 | 0.003 | 29606 | 0.045 |
| SSRGD8699 | SSRGD17837 | 0.023 | 0.001 | 42288 | 0.036 | 0.045 | 0.003 | 34995 | 0.064 |
| SSRGD8699 | SSRGD8997 | 0.022 | 0.001 | 42476 | 0.036 | 0.000 | 0.000 | 37640 | 0.000 |
| SSRGD8997 | SSRGD13517 | 0.201 | 0.001 | 50225 | 0.227 | 0.000 | 0.000 | 35343 | 0.000 |
| SSRGD8997 | SSRGD17837 | 0.022 | 0.001 | 42919 | 0.036 | 0.000 | 0.000 | 42443 | 0.000 |

**Table S3.** H-W equilibrium test for each *loci* in the eight populations of *G. decorticans*.

|  |  | **HWTEST** | | | | |
| --- | --- | --- | --- | --- | --- | --- |
| **Pop** | **Locus** | **DF** | **ChiSq** | **Pval** | **Signif** | **Signif B** |
| Azapa | SSRGD27558 | 15 | 45.209 | 0.000 | *** | *** |
|  | SSRGD11258 | 6 | 6.237 | 0.397 | ns | ns |
|  | SSRGD7345 | 3 | 22.516 | 0.000 | *** | *** |
|  | SSRGD17951 | 15 | 54.993 | 0.000 | *** | *** |
|  | SSRGD11733 | 15 | 27.683 | 0.024 | * | ns |
|  | SSRGD8699 | 10 | 93.213 | 0.000 | *** | *** |
|  | SSRGD8997 | 10 | 24.651 | 0.006 | ** | ns |
|  | SSRGD17837 | 36 | 139.881 | 0.000 | *** | *** |
|  | SSRGD13517 | 21 | 56.807 | 0.000 | *** | *** |
| Calama | SSRGD27558 | 1 | 0.194 | 0.659 | ns | ns |
|  | SSRGD11258 | 3 | 7.778 | 0.051 | ns | ns |
|  | SSRGD7345 | 6 | 21.000 | 0.002 | ** | ns |
|  | SSRGD17951 | 3 | 14.000 | 0.003 | ** | ns |
|  | SSRGD11733 | 15 | 25.667 | 0.042 | * | ns |
|  | SSRGD8699 | 1 | 0.194 | 0.659 | ns | ns |
|  | SSRGD8997 | 10 | 22.286 | 0.014 | * | ns |
|  | SSRGD17837 | 3 | 7.000 | 0.072 | ns | ns |
|  | SSRGD13517 | 6 | 12.833 | 0.046 | * | ns |
| Chaca | SSRGD27558 | 3 | 3.220 | 0.359 | ns | ns |
|  | SSRGD11258 | 15 | 12.164 | 0.667 | ns | ns |
|  | SSRGD7345 | 6 | 9.017 | 0.173 | ns | ns |
|  | SSRGD17951 | 15 | 35.655 | 0.002 | ** | ns |
|  | SSRGD11733 | 21 | 24.193 | 0.284 | ns | ns |
|  | SSRGD8699 | 15 | 17.011 | 0.318 | ns | ns |
|  | SSRGD8997 | 6 | 22.057 | 0.001 | ** | ns |
|  | SSRGD17837 | 10 | 16.753 | 0.080 | ns | ns |
|  | SSRGD13517 | 21 | 31.944 | 0.059 | ns | ns |
| Copiapo | SSRGD27558 | 66 | 92.608 | 0.017 | * | ns |
|  | SSRGD11258 | 55 | 68.333 | 0.107 | ns | ns |
|  | SSRGD7345 | 6 | 10.717 | 0.098 | ns | ns |
|  | SSRGD17951 | 28 | 45.341 | 0.020 | * | ns |
|  | SSRGD11733 | 36 | 86.271 | 0.000 | *** | *** |
|  | SSRGD8699 | 15 | 14.139 | 0.515 | ns | ns |
|  | SSRGD8997 | 36 | 63.978 | 0.003 | ** | ns |
|  | SSRGD17837 | 153 | 209.788 | 0.002 | ** | ns |
|  | SSRGD13517 | 10 | 8.807 | 0.550 | ns | ns |
| Coquimbo | SSRGD27558 | 21 | 71.250 | 0.000 | *** | *** |
|  | SSRGD11258 | 28 | 64.262 | 0.000 | *** | *** |
|  | SSRGD7345 | 3 | 19.000 | 0.000 | *** | *** |
|  | SSRGD17951 | 10 | 19.000 | 0.040 | * | ns |
|  | SSRGD11733 | 21 | 44.735 | 0.002 | ** | ns |
|  | SSRGD8699 | 6 | 16.382 | 0.012 | * | ns |
|  | SSRGD8997 | 6 | 19.411 | 0.004 | ** | ns |
|  | SSRGD17837 | 10 | 18.829 | 0.042 | * | ns |
|  | SSRGD13517 | 6 | 1.830 | 0.935 | ns | ns |
| Pachica | SSRGD27558 | Monomorphic |  |  |  |  |
|  | SSRGD11258 | 10 | 53.288 | 0.000 | *** | *** |
|  | SSRGD7345 | 1 | 25.000 | 0.000 | *** | *** |
|  | SSRGD17951 | 1 | 25.000 | 0.000 | *** | *** |
|  | SSRGD11733 | 6 | 16.335 | 0.012 | * | ns |
|  | SSRGD8699 | 1 | 25.000 | 0.000 | *** | *** |
|  | SSRGD8997 | 6 | 32.823 | 0.000 | *** | *** |
|  | SSRGD17837 | 15 | 75.000 | 0.000 | *** | *** |
|  | SSRGD13517 | 10 | 33.239 | 0.000 | *** | *** |
| Pica | SSRGD27558 | 10 | 40.000 | 0.000 | *** | *** |
|  | SSRGD11258 | 21 | 19.792 | 0.534 | ns | ns |
|  | SSRGD7345 | Monomorphic |  |  |  |  |
|  | SSRGD17951 | 3 | 9.446 | 0.024 | * | ns |
|  | SSRGD11733 | 3 | 6.694 | 0.082 | ns | ns |
|  | SSRGD8699 | 3 | 0.123 | 0.989 | ns | ns |
|  | SSRGD8997 | 6 | 10.000 | 0.125 | ns | ns |
|  | SSRGD17837 | 6 | 21.111 | 0.002 | ** | ns |
|  | SSRGD13517 | 6 | 30.000 | 0.000 | *** | *** |
| San Pedro | SSRGD27558 | 6 | 23.356 | 0.001 | *** | ns |
|  | SSRGD11258 | 36 | 51.140 | 0.049 | * | ns |
|  | SSRGD7345 | 3 | 3.059 | 0.383 | ns | ns |
|  | SSRGD17951 | 10 | 50.917 | 0.000 | *** | *** |
|  | SSRGD11733 | 105 | 185.451 | 0.000 | *** | *** |
|  | SSRGD8699 | 15 | 37.686 | 0.001. | ** | ns |
|  | SSRGD8997 | 15 | 81.607 | 0.000 | *** | *** |
|  | SSRGD17837 | 15 | 97.261 | 0.000 | *** | *** |
|  | SSRGD13517 | 10 | 3.823 | 0.955 | ns | ns |

**Note:** signif B is the significance for the Bonferroni correction for multiple tests; ns= not significant, * P< 0.05. ** P< 0.01. *** P< 0.001
